# MolGlueDB: an online database of molecular glues

**DOI:** 10.1093/nar/gkaf811

**Published:** 2025-08-21

**Authors:** Xiao Wang, Zhiyao Zhuang, Chengwei Zhang, Bowen Zhang, Wei Zhan, Yifan Wang, Zhaojuan Liu, Shanwen Yuan, Wenjia Niu, Qi He, Yanqing Tian, Ximing Xu, Senbiao Fang, Chong Qin

**Affiliations:** Key Laboratory of Marine Drugs, Chinese Ministry of Education, School of Medicine and Pharmacy, Ocean University of China, Qingdao, Shandong 266003, China; Center for Targeted Protein Degradation and Drug Discovery, Ocean University of China, Qingdao, Shandong 266003, China; Key Laboratory of Marine Drugs, Chinese Ministry of Education, School of Medicine and Pharmacy, Ocean University of China, Qingdao, Shandong 266003, China; Key Laboratory of Marine Drugs, Chinese Ministry of Education, School of Medicine and Pharmacy, Ocean University of China, Qingdao, Shandong 266003, China; Key Laboratory of Marine Drugs, Chinese Ministry of Education, School of Medicine and Pharmacy, Ocean University of China, Qingdao, Shandong 266003, China; Key Laboratory of Marine Drugs, Chinese Ministry of Education, School of Medicine and Pharmacy, Ocean University of China, Qingdao, Shandong 266003, China; Key Laboratory of Marine Drugs, Chinese Ministry of Education, School of Medicine and Pharmacy, Ocean University of China, Qingdao, Shandong 266003, China; Key Laboratory of Marine Drugs, Chinese Ministry of Education, School of Medicine and Pharmacy, Ocean University of China, Qingdao, Shandong 266003, China; Key Laboratory of Marine Drugs, Chinese Ministry of Education, School of Medicine and Pharmacy, Ocean University of China, Qingdao, Shandong 266003, China; Key Laboratory of Marine Drugs, Chinese Ministry of Education, School of Medicine and Pharmacy, Ocean University of China, Qingdao, Shandong 266003, China; Key Laboratory of Marine Drugs, Chinese Ministry of Education, School of Medicine and Pharmacy, Ocean University of China, Qingdao, Shandong 266003, China; Key Laboratory of Marine Drugs, Chinese Ministry of Education, School of Medicine and Pharmacy, Ocean University of China, Qingdao, Shandong 266003, China; Key Laboratory of Marine Drugs, Chinese Ministry of Education, School of Medicine and Pharmacy, Ocean University of China, Qingdao, Shandong 266003, China; Marine Biomedical Research Institute of Qingdao, Qingdao, Shandong 266071, China; Center for Targeted Protein Degradation and Drug Discovery, Ocean University of China, Qingdao, Shandong 266003, China; Marine Biomedical Research Institute of Qingdao, Qingdao, Shandong 266071, China; Key Laboratory of Marine Drugs, Chinese Ministry of Education, School of Medicine and Pharmacy, Ocean University of China, Qingdao, Shandong 266003, China; Center for Targeted Protein Degradation and Drug Discovery, Ocean University of China, Qingdao, Shandong 266003, China; Marine Biomedical Research Institute of Qingdao, Qingdao, Shandong 266071, China; Laboratory for Marine Drugs and Bioproducts, Qingdao Marine Science and Technology Center, Qingdao, Shandong 266137, China

## Abstract

Molecular glue degraders (MGDs) and proteolysis-targeting chimeras (PROTACs) are two prominent approaches in targeted protein degradation, both leveraging the ubiquitin-proteasome system to induce selective protein degradation. While PROTACs rely on heterobifunctional motifs, MGDs possess more compact, mono-affinitive molecular structures, offering distinct advantages in drug-like properties. Over the past decade, significant progress has been made in development of MGDs, with over 20 MGDs advancing through clinical trials. Despite these advancements, the field remains in its early stages due to the novel mode of action and complex design principles. To facilitate rational design of MGDs, we present MolGlueDB, an open-access, web-based database consolidating information from 241 publications (January 2001–May 2025). MolGlueDB contains 1840 entries, including 1629 distinct MGDs, 28 recruiting proteins, and 94 targets, with comprehensive data on chemical structures, binding affinities, degradation capacities, biological activities, and physicochemical properties. The platform supports both text-based and structure-based searches, enabling users to refine results based on specific molecular attributes. MolGlueDB is freely accessible at https://www.molgluedb.com, providing a valuable resource for advancing research of MGDs and accelerating drug discovery in the field of targeted protein degradation.

## Graphical Abstract

**Graphical Abstract:**
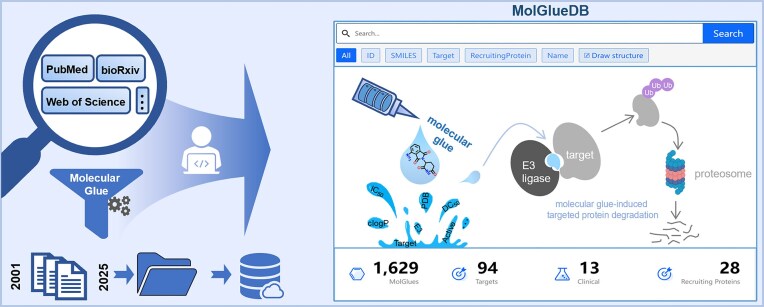

## Introduction

In recent years, targeted protein degradation has emerged as a promising strategy in innovative therapeutics discovery and development, with proteolysis-targeting chimeras (PROTACs) and molecular glue degraders (MGDs) standing out as its most prominent approaches. Though mechanistically distinct, they complement each other by expanding the landscape of degradable targets and overcoming the limitations of traditional small-molecule inhibitors [1–3].

MGDs are usually monovalent small molecules (∼500 Da) that bind to E3 ligases, such as CRBN, and modify their binding surfaces to reprogram interactions with substrate proteins. By reshaping the binding specificity of scaffolding proteins, MGDs induce novel protein–protein interactions (PPIs) [4], ultimately promoting target protein degradation through the ubiquitin-proteasome system. This unique mechanism enables the elimination of previously undruggable proteins, offering an alternative approach for targeted therapy. Compared to PROTACs, MGDs exhibit distinct biological properties and advantageous physicochemical characteristics [5, 6], including smaller molecular size, enhanced membrane permeability, and greater oral bioavailability, as they are more likely to comply with Lipinski’s Rule of Five. Furthermore, their compact structures facilitate superior blood-brain barrier penetration [7], expanding their therapeutic potential across a broader range of diseases. Thus, MGDs represent powerful and complementary approaches alongside PROTACs in targeted protein degradation, reinforcing the roles of MGDs in the next generation of innovative drug discovery.

**Figure 1.**
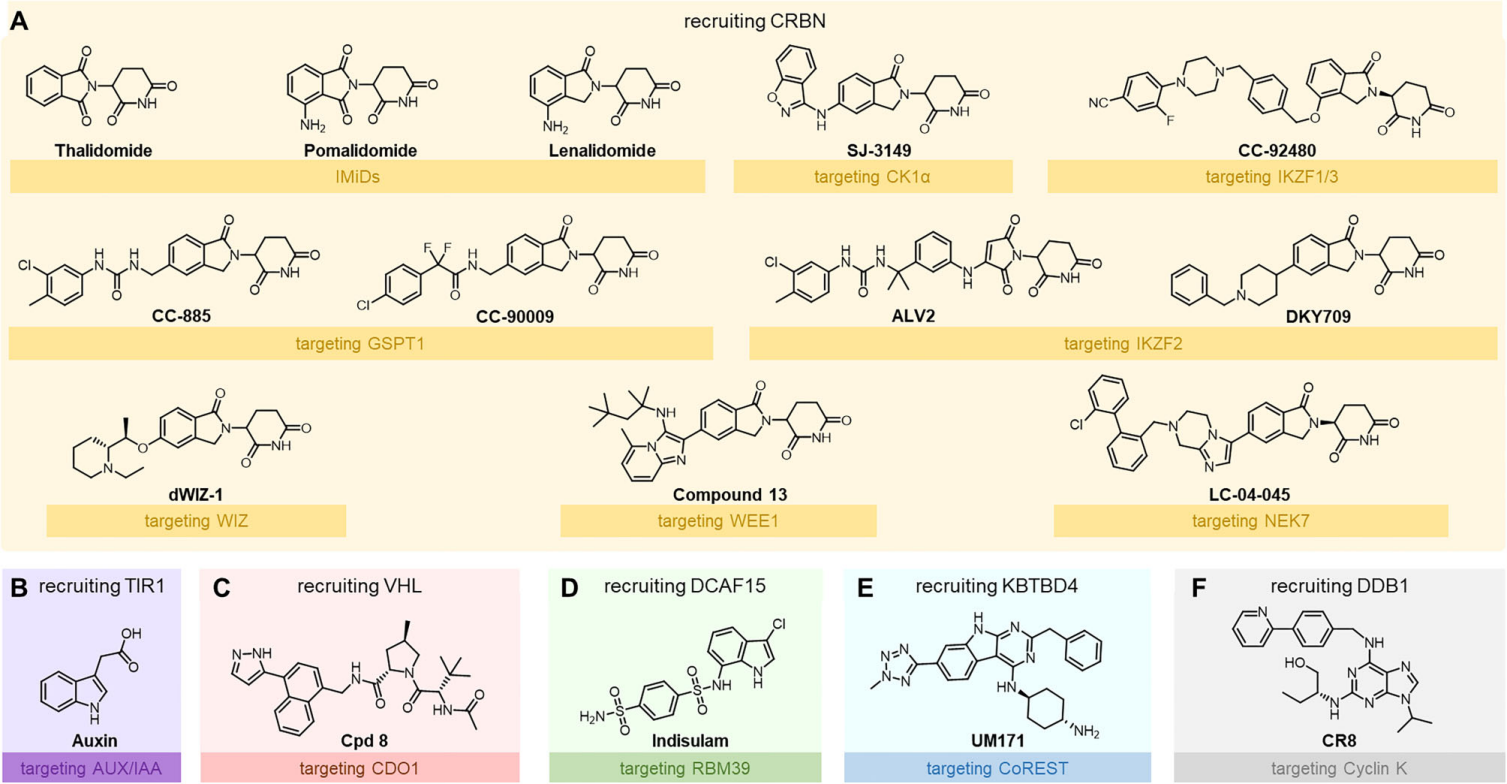
(**A**–**F**) Representative MGDs recruiting E3 ligase substrate receptors, including TIR1, CRBN, VHL, DCAF15, KBTBD4, and E3 ligase adaptor DDB1. Targets of these MGDs are highlighted.

In the 1950s and 1960s, thalidomide (Fig. 1A) was developed and widely prescribed as an antiemetic but was soon withdrawn after causing severe congenital malformations, notably phocomelia. Subsequent studies on thalidomide and its analogs, lenalidomide (approved in 2005) and pomalidomide (approved in 2013), revealed their potent immunomodulatory and anti-myeloma activities as immunomodulatory drugs (IMiDs) [8], spurring investigations into their molecular mechanisms. In 2010, Ito *et al.* [9] identified cereblon (CRBN) as thalidomide’s direct binding target and a substrate receptor within the Cul4–RBX1–DDB1 (CRL4) E3 ubiquitin ligase complex. Binding of thalidomide modulates the complex’s ubiquitination activity, potentially explaining its teratogenic effects. In 2014, Fischer *et al.* [10] elucidated the binding mode of the three IMiDs with CRBN and proposed the formation of the CRL4^CRBN^–IMiD–IKZF1/3 ternary complex to effect IKZF1/3 degradation via a molecular glue mechanism. This landmark research significantly promoted the structural biology-guided discovery of CRBN-based MGDs [11–23].

Notably, the concept of MGD predates the elucidation of thalidomide’s mechanism. As early as 2001, Gray *et al.* [24] identified the plant hormone auxin (Fig. 1B) as an AUX/IAA degrader that recruits E3 ligase TIR1. Later in 2007, Zheng’s group [25] resolved the TIR1–auxin–AUX/IAA ternary complex structure and elucidated the mechanistic basis of auxin-induced degradation. This work reintroduced the concept of “molecular glue” (MG), a term originally coined by Schreiber in the 1990s [26], and provided the first detailed mechanistic insight into MGDs. Thus, auxin is considered the first fully characterized MGD, although thalidomide remains the first synthetic example.

Recently, the scope of MGDs has expanded beyond CRBN to include alternative E3 ligases—more precisely, E3 substrate receptor proteins—such as DCAF15 [27], VHL [28], and KBTBD4 [29, 30] (Fig. 1C–E). Notably, MGD CR8 (Fig. 1F) was reported to induce Cyclin K degradation by binding CDK12 [31], forming an artificial PPI interface that enables direct CR8–CDK12 association with DDB1 and forming a CRL4–CR8–CDK12–Cyclin K complex. Within the CRL4 E3 ligase complex, DDB1 functions as an adaptor bridging the Cullin scaffold and DCAF substrate receptors without determining substrate specificity. In this case, CR8’s bridging mechanism bypasses the need for a canonical DCAF substrate receptor, although the degradation still depends on core Cullin-RING E3 ligase components.

**Figure 2.**
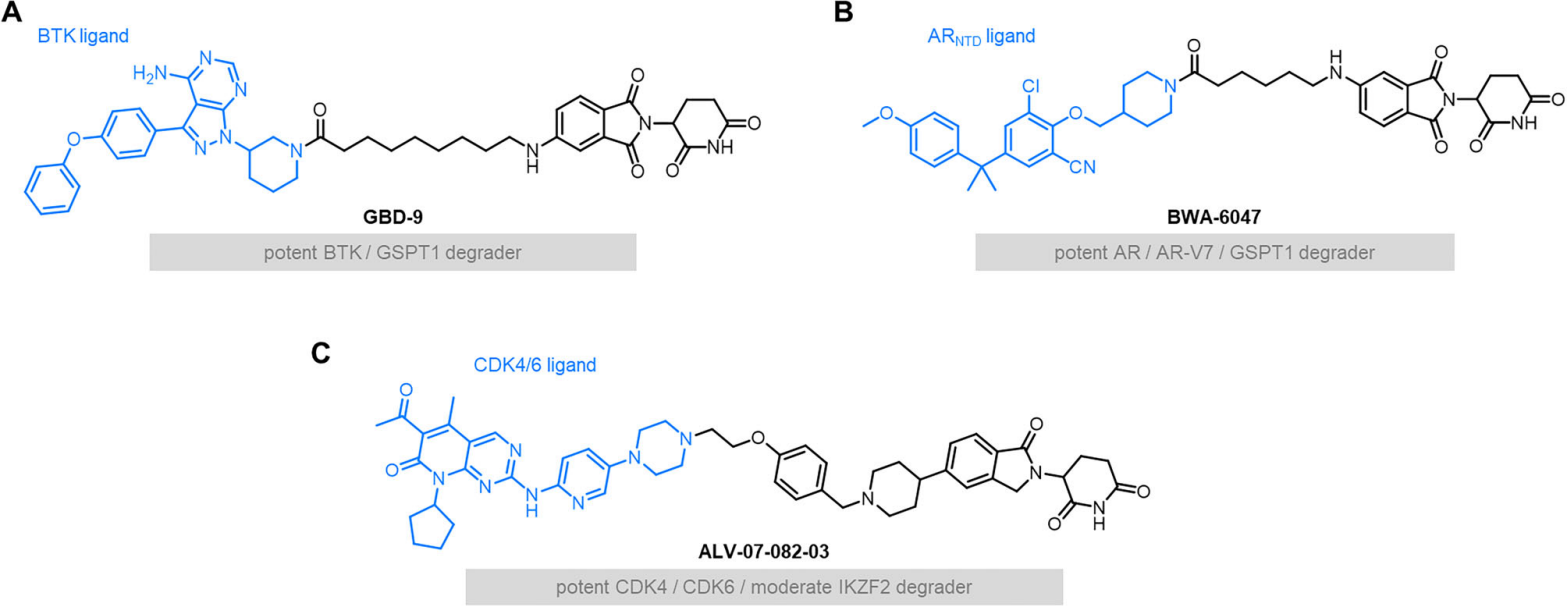
Representative PROTACs exhibiting MGD-like properties. (**A**, **B**) Structures of PROTACs GBD-9 and BWA-6047, both featuring flexible linkers that conjugate protein-of-interest (POI) ligands (highlighted in blue) to thalidomide-based CRBN ligands. (**C**) AL-07-082-03, a triple degrader targeting CDK4, CDK6, and IKZF2, generated by linking the IKZF2-targeting MGD DKY709 to a CDK4/6 ligand.

Recent studies have shown that CRBN-recruiting PROTACs can also function as MGDs, inducing degradation of neo-substrates like GSPT1 [32] and zinc finger proteins [33]. While these off-target effects raise safety concerns [34, 35], they can offer therapeutic advantages when degradation of neo-substrates and PROTAC targets produce synergistic anti-cancer effects. For instance (Fig. 2A and B), BTK/GSPT1 dual degrader GBD-9 from Rao’s group [36] and our group’s AR/AR-V7/GSPT1 triple degrader BWA-6047 [37] showed potent synergy. Alternatively, Gray’s group [38] linked the IKZF2-targeting MGD DKY709 to a CDK4/6 ligand to create AL-07-082-03 (Fig. 2C), which efficiently degraded IKZF2, CDK4, and CDK6. These examples highlight how PROTACs incorporating IMiD-based CRBN recruiters can achieve MGD-like neo-substrate degradation with therapeutic potential.

To date, over 20 MGDs have entered clinical trials [39], including approved drugs (thalidomide, lenalidomide, and pomalidomide), as well as IKZF1/3 degraders in Phase III trials—CC-220, CC-92480, and CC-99282 (NCT05827016, NCT05552976, NCT06356129). In comparison, ∼30 PROTACs have advanced to clinical evaluation [40] over more than two decades of development, with two reaching Phase III (BGB-16673 targeting BTK, NCT06846671; and BMS-986365 targeting AR, NCT06764485). The most advanced PROTAC, ARV-471 targeting ER, recently submitted its New Drug Application (NDA) to the FDA on 6 June 2025, but has not yet been approved. These comparisons demonstrate that MGDs have achieved comparable, or even faster, clinical advancement within a shorter timeframe than PROTACs, despite the latter being mechanistically elucidated earlier and generally more amenable to rational design.

However, MGDs still face several challenges in therapeutic development [3, 41–43]. Most MGDs rely on CRBN, while the potential of >600 human E3 ligases remains unexplored. Expanding E3 pool and elucidating their substrate specificity are critical for diversifying MGDs’ applications. Furthermore, most MGDs are discovered serendipitously, with incompletely understood mechanisms. A single MGD–E3 pair may engage diverse targets via ternary complexes, yet predicting or controlling PPI selectivity remains difficult. Although preliminary models for predicting ternary complexes have been reported [44–46], their accuracy and generalizability are still limited. Additionally, not all MGD candidates trigger effective degradation, while some act as non-degradative MGs. Structural optimization is further hindered by complex PPIs, limited scaffolds, and poorly characterized pharmacokinetic (PK) and pharmacodynamic (PD) profiles.

To overcome these limitations, integrative approaches combining structural biology, medicinal chemistry, and advanced screening methodologies are essential. Most importantly, leveraging the wealth of reported MGD structures and activity data to uncover key correlations, establish rational design principles, and gain deeper insights into emerging trends will be essential for advancing the field.

Herein, we developed MolGlueDB, the first dedicated online database for MGDs. MolGlueDB integrates comprehensive information, including chemical structures, physicochemical properties, targets, binding affinities, biological activities, ADMET profiles, therapeutic indications, and proteomics data. It supports text- and structure-based queries, and data export in SDF/CSV formats. Freely accessible at https://www.molgluedb.com, MolGlueDB is designed to accelerate MGD discovery, mechanistic studies, and rational design.

## Materials and methods

### Criteria for molecules collected in the database

**Figure 3.**
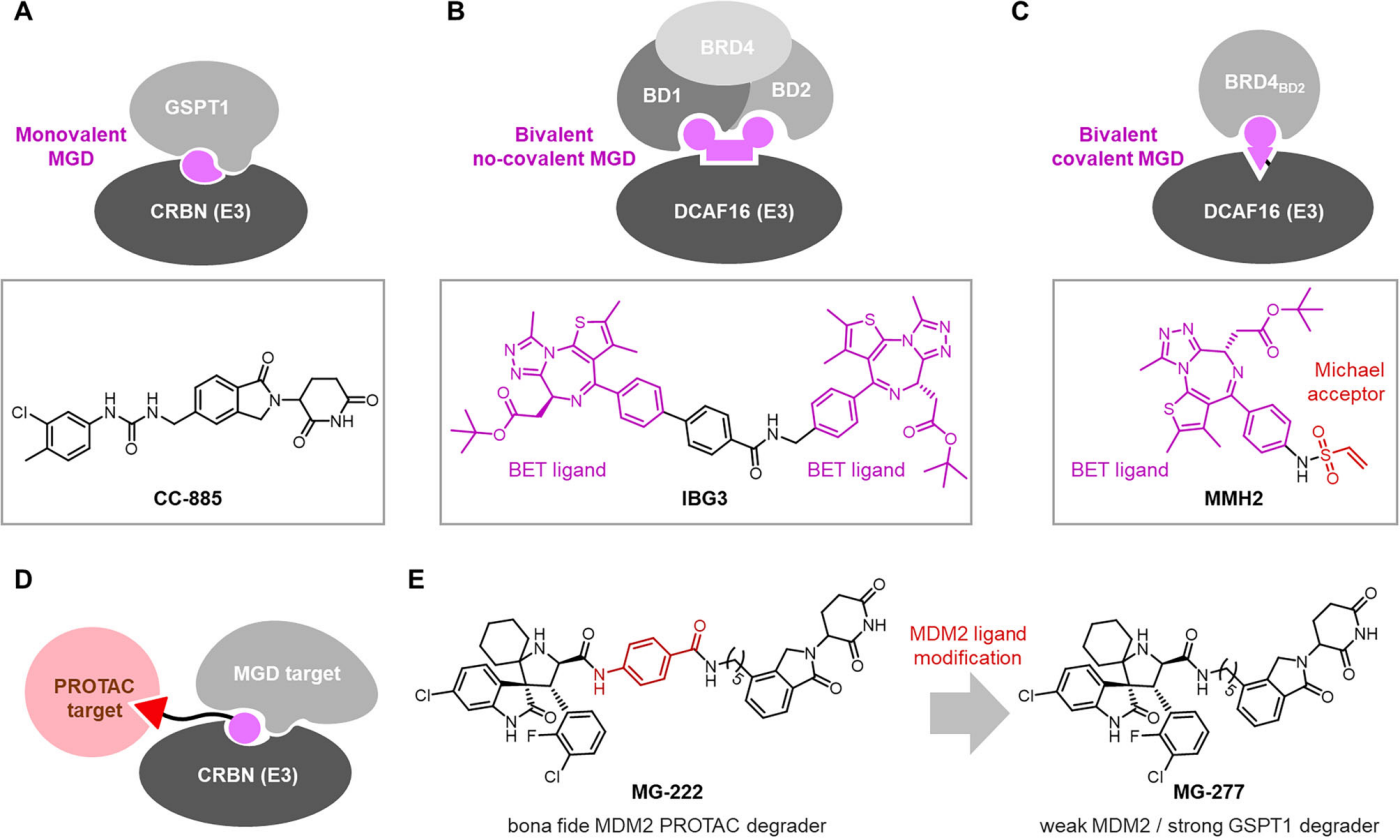
Mechanistic schematics and representative examples of different MGD types and MGD-like PROTACs. (**A**) Classical monovalent MGDs. (**B**) Bivalent non-covalent MGDs. (**C**) Bivalent covalent MGDs. (**D**) PROTACs with MGD-like properties. (**E**) Schematic illustration showing conversion of bona fide MDM2 PROTAC MG-222 into pure MGD MG-277 through subtle structural modification.

In this study, MGDs are defined to include:

(a)*Classical monovalent MGDs*. Historically, an MGD refers to mono-affinitive small molecule that binds an E3 ligase and remodels its surface to recruit a neo-substrate via induced PPIs (Fig. 3A). In addition, some mono-affinitive molecules that bind non-E3 proteins, such as CR8 described earlier in Fig. 1F, can similarly promote E3–MGD–substrate complex formation and trigger substrate degradation. All such mono-affinitive degraders are classified as monovalent MGDs in this database.

(b) *Bivalent non-covalent/covalent MGDs*. Recent studies have expanded the definition of MGDs to include bivalent molecules that simultaneously engage two distinct domains, either within the target protein or between the target and the E3 ligase. For example, IBG3, reported by Winter and Ciulli’s team [47], tethers two BET ligands to engage both BD1 and BD2 domains of BRD4 while recruiting DCAF16 (Fig. 3B), leading to BRD4 degradation via a molecular glue mechanism. Additionally, covalent bivalent MGDs such as MMH2, reported by Gray, Fischer, and Ebert’s team [48], combine the BET ligand with an electrophilic Michael acceptor. MMH2 first binds BRD4_BD2_, which orients the Michael warhead for nucleophilic attack by DCAF16 Cys58, resulting in covalent modification (Fig. 3C) that stabilizes the ternary complex and promotes BRD4 degradation. Notably, its bivalent nature is supported by the pronounced hook effect observed. These novel covalent bivalent MGDs are also included in the database.

(c) *MGD candidates without degradation activity*. During the design and bioevaluation of potent MGDs targeting specific proteins, many candidates exhibit minimal or no degradation activity against the intended substrate. Although these molecules fail to induce degradation, they retain valuable structure–activity relationship (SAR) information. Moreover, given the frequent multi-target nature of MGDs, candidates reported as inactive against a particular tested substrate may still possess degradative activity toward other untested targets. Accordingly, these molecules are included in the database as potential scaffolds for design of MGDs.

(d) *PROTACs with reported MGD-like behaviors*. Some CRBN-recruiting PROTACs, in addition to degrading their intended POI targets, can modulate the CRBN surface to recruit and degrade neo-substrates (Figs 2 and 3D). While such neo-substrate degradation raises concerns about potential off-target toxicity, it can also confer synergistic pharmacological effects when the PROTAC’s bona fide target and the neo-substrate cooperate in disease modulation. Moreover, structure-guided modifications can convert a bona fide PROTAC into a pure MGD, as exemplified by MG-277, which is derived from the MDM2 PROTAC MG-222 through subtle structural changes to its MDM2 ligand moiety (Fig. 3E), resulting in losing most of its MDM2-degrading activity but gaining potent GSPT1 degradation as an MGD [49]. Collecting such PROTACs in this database will facilitate deeper understanding of the continuum between PROTACs and MGDs, enabling rational design of PROTACs to either enhance MGD-like synergistic effects or reduce off-target liabilities for improved selectivity.

### Future inclusion of non-degradative molecular glues in MolGlueDB

Currently, degradative MGs (i.e. MGDs) remain the primary focus of research, whereas non-degradative MGs are far less explored. It is important to note that non-degradative MGs differ fundamentally from the inactive MGD candidates described above. Inactive MGD candidates are compounds designed specifically for degradation but found to lack such activity upon experimental evaluation; they are not assessed for non-degradative glue functions. In contrast, non-degradative MGs modulate target function by forming ternary complexes without inducing degradation. Notable examples of non-degradative MGs include classical natural products such as FK506, rapamycin, and cyclosporin A, as well as recent KRAS-targeting glues from Revolution Medicines (e.g. RMC-6291, RMC-9805, and RMC-6236) [50, 51]. Given the current research landscape, MolGlueDB prioritized degradative MGs in its initial release to comprehensively cover the most extensively studied class to date. Nonetheless, we are actively monitoring advances in non-degradative MGs and have already scheduled their inclusion in *future major updates*.

### Data collection, processing, and database construction

**Figure 4.**
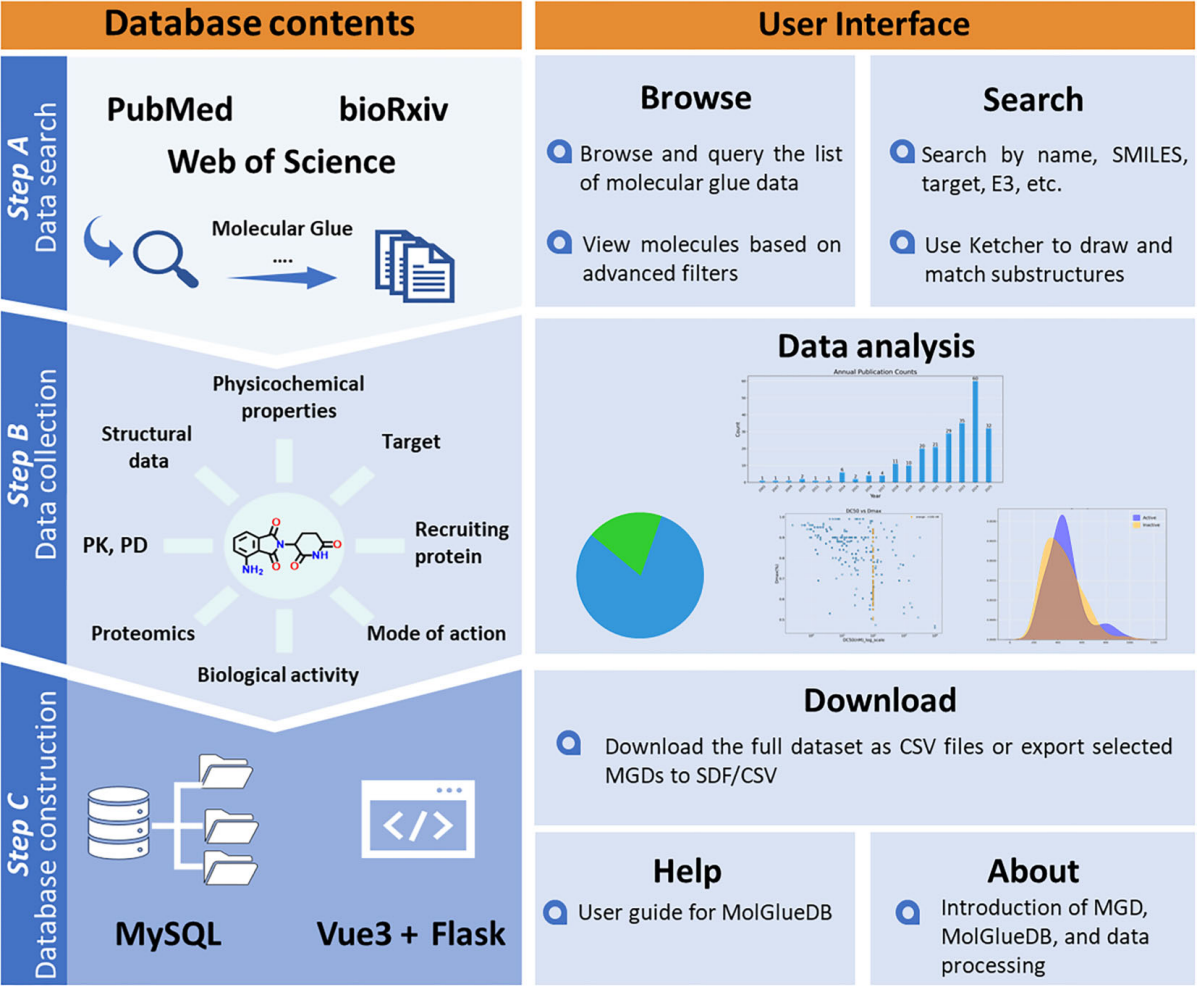
Overview of MolGlueDB: construction workflow and core features of database and user interface.

As illustrated in Fig. 4, MGD-related literature was systematically collected and curated in three steps. Step A, data collection: keyword searches using “molecular glue”, “MG”, or “CRBN” were used to search Google, Google Scholar, Web of Science, PubMed, and bioRxiv. Each retrieved article underwent manual screening to exclude inaccessible full texts, non-research articles (e.g. reviews, news, perspectives, posters, meeting abstracts), computational-only studies lacking experimental validation of activity or new ternary complex structures, and studies unrelated to degradative MGDs. Subsequently, references cited within eligible articles and relevant review papers were cross-checked to identify and incorporate any missing MGDs, ultimately yielding 241 relevant articles across 79 journals.

Step B, data processing: a comprehensive parameter framework was defined to ensure uniformity and depth of coverage. Key extracted information included structure (core scaffolds and pharmacophores), nomenclature, biological activity (e.g. degradation potency, western blot validation, and antiproliferative activity), target, recruiting proteins, mode of action (covalent or non-covalent), binding affinities (e.g. IC_50_, EC_50_, and *K*_d_ parameters of HTRF, TR-FRET, SPR, and FP assays), binary/ternary complex structural data (X-ray or cryo-EM), *in vivo* PK/PD data, and proteomics results. To standardize and accelerate this process, a customized C# program was developed to facilitate data entry and integrate RDKit for automated calculation of molecular physicochemical properties. Each publication was systematically annotated to generate a structured and analyzable dataset, which further underwent rigorous cleansing to remove invalid characters, revise corrupted entries, and standardize field formats. This was implemented through automated Python scripts combined with iterative manual reviews to ensure data integrity and accuracy. The curated data were organized into three major datasets: (a) general information, (b) biological assay and experimental data, and (c) calculated physicochemical properties (see Supplementary Table S1). These were subsequently structured into a MySQL database to enable robust back-end storage and querying.

Step C, database construction: For website implementation, MolGlueDB was built with a modern Vue 3 frontend and Python Flask backend connected to the MySQL 8 database, and deployed on a Linux server with Nginx for stability and scalability. Molecular structures were rendered using RDKit from SMILES codes, while the Ketcher editor (https://github.com/epam/ketcher) enabled interactive drawing and substructure searches.

Within MolGlueDB, DC_50_ (concentration causing 50% degradation) and D_max_ (maximum degradation level) were systematically collected to quantify the degradation capacity of MGDs; where unavailable, reported degradation percentages were used. Given the broad substrate degradation profiles commonly observed for MGDs, the database classifies targets with strong degradation activity (typically with DC_50_ in the low hundreds of nanomolar range) as primary targets, while secondary targets show weaker degradation (generally above this range). If a given MGD does not exhibit degradation activity of any target in this range, the most significantly degraded target is assigned as its primary target(s), and no secondary target is specified. For a PROTAC degrader exhibiting MGD-like behavior, only the substrate protein degraded via molecular glue activity is recorded as the primary target, rather than the target corresponding to the PROTAC’s original POI ligand.

Ultimately, MolGlueDB comprises 1840 entries, including 1629 distinct MGDs, covering 94 primary and secondary targets and 28 recruiting proteins with comprehensive annotations. The MolGlueDB website provides an intuitive user interface supporting functionalities that include browsing all molecular glue entries with detailed properties, text-based searches (by name, SMILES, MolGlueDB ID, target, or E3 ligase), and substructure searches integrated with the Ketcher editor.

To facilitate data interpretation, the website integrates multiple quantitative visualization modules, including annual publication trends (2001–2025), activity status distributions, degradation and antiproliferation scatter plots (e.g. log_10_DC_50_ versus D_max_; log_50_DC_50_ versus log_10_IC_50_), and density plots of key Lipinski’s Rule of Five parameters (molecular weight, tPSA, clogP, HBD/HBA counts) (Supplementary Fig. S1). Multi-dimensional Venn diagram analyses further reveal data coverage overlaps among affinity, protein–small molecule complex structures, proteomics, degradation information, and antiproliferation datasets at MGD, recruiting protein, and target levels (Supplementary Fig. S2). Additionally, interactive sunburst charts summarize binary and ternary complex structures by recruiting protein, target, and MolGlueDB entry, with direct links to RCSB PDB and EMDB resources for detailed structural exploration (Supplementary Fig. S3).

To facilitate data utilization, MolGlueDB offers multiple export options, including full-table CSV downloads and SDF/CSV exports for selected entries. Moreover, user guides and an introductory overview of MGDs are provided to maximize accessibility and support for the research community. Collectively, these features provide comprehensive insights into the structural, functional, and pharmacological characteristics of MGDs curated in MolGlueDB.

## Results

### Searching and browsing

#### Searching

**Figure 5.**
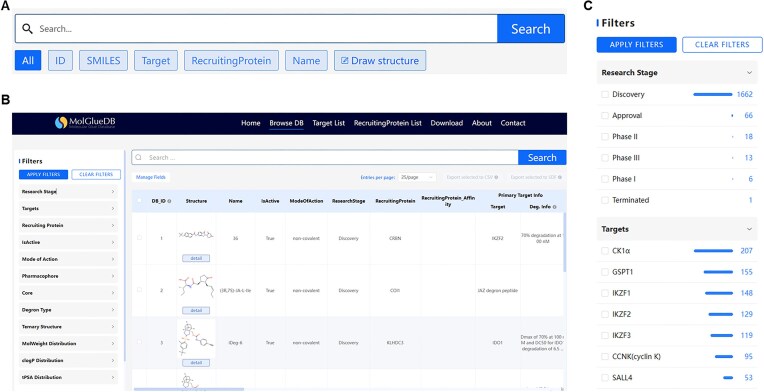
Search and browsing features of MolGlueDB. (**A**) Configuration of the search box. (**B**) Main database interface showing advanced filters on the left, search box at the top right, and data table below. (**C**) Expanded view of the advanced filters, displaying available filtering options.

MolGlueDB supports both text-based and structure-based searches to meet diverse user needs. Users can perform quick lookups using keywords such as ID for the database, SMILES, and target names, or conduct advanced substructure searches by sketching or uploading molecular structures via the integrated Ketcher editor. Substructure matching is powered by RDKit, enabling precise identification of structurally related compounds (Fig. 5A).

#### Browsing

MolGlueDB provides an integrated “Browse DB” interface (Fig. 5B), where all entries are presented in a structured, searchable table. Users can perform multi-parameter filtering and keyword-based searching simultaneously to refine query results. Available filters cover key dimensions such as research stage, target identity, biological activity, structural features, ternary complex availability, and physicochemical properties (Fig. 5C). In addition, key physicochemical properties, such as MolWeight, tPSA, clogP, logS, HBA/HBD Count, and Rotatable Bond Count, are directly sortable via a tri-state column header toggle (ascending, descending, or reset). This interactive design streamlines compound comparison and facilitates efficient dataset exploration.

**Figure 6.**
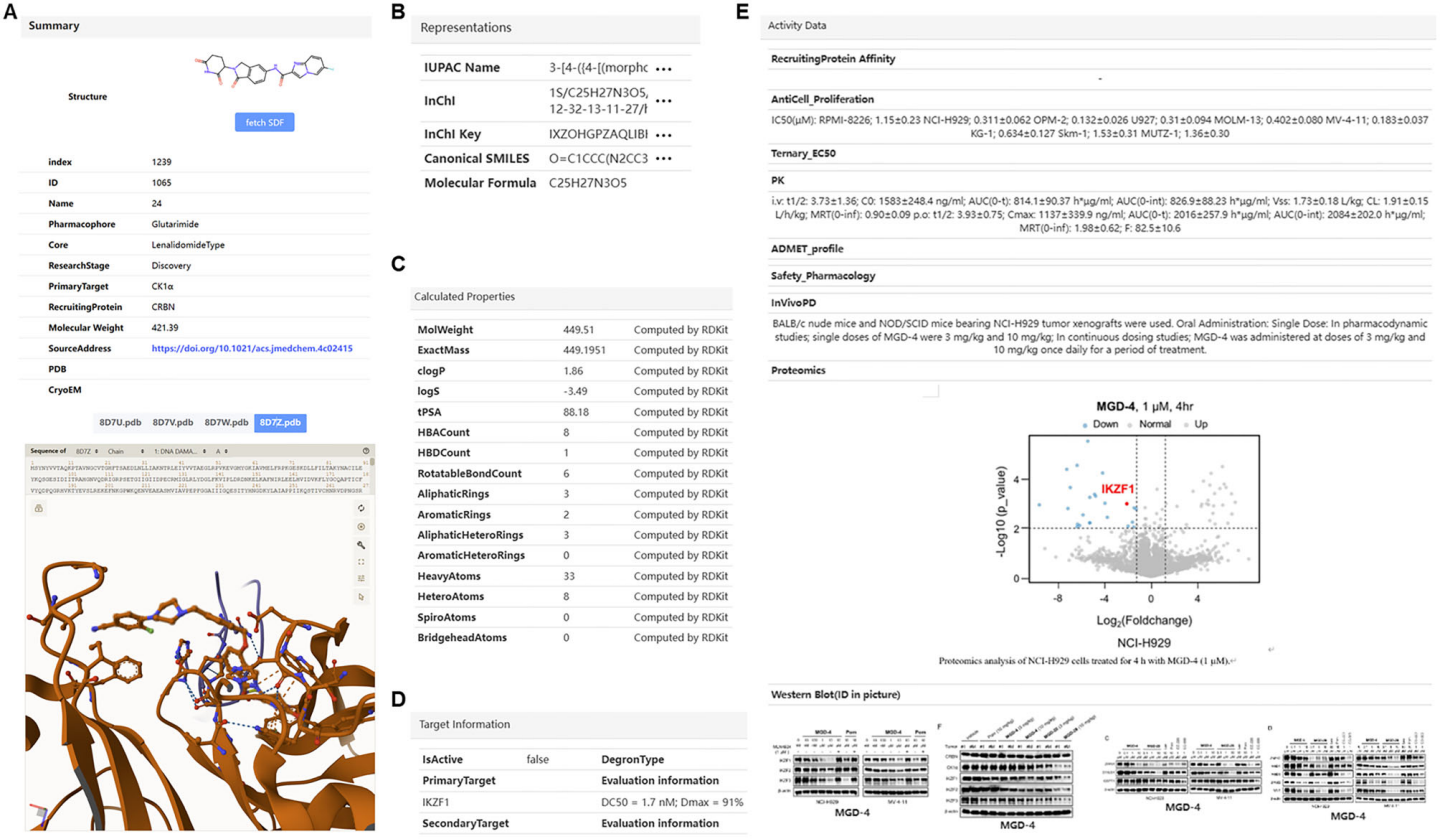
Compound detail page interface. (**A**) Summary section with embedded interactive Mol* viewer displaying available PDB complex structures. (**B**) Representations. (**C**) Calculated properties. (**D**) Target information. (**E**) Activity data tabs. Images shown are assembled from different entries.

To enhance usability, each compound entry includes a “detail” button under its structure image that redirects users to its detailed information page. These pages integrate molecular representations, calculated and experimental physicochemical properties, target and degradation data, biological activity profiles, and supporting evidence such as western blot and proteomics images (Fig. 6A–E). Additionally, these pages embed the open-source viewer Mol* (molstar, Fig. 6A), enabling interactive visualization and analysis of available PDB complex structures. Collectively, these well-organized pages provide a comprehensive view of each compound’s characteristics and research context.

By integrating diverse functionalities and a robust analytical framework, MolGlueDB is designed to meet the increasing demands for data-driven research in targeted protein degradation. By enabling systematic characterization and efficient data exploration, it aims to accelerate therapeutic discoveries and optimize drug design strategies for MGDs.

### Future development plans

To ensure data accuracy and functional improvements, MolGlueDB is committed to continuous updates and optimizations:

- *Quarterly database updates*: The database will be updated every three months with newly reported MGDs and experimental data from research articles published since the previous update.
- *Annual structural and UI enhancements*: To ensure optimal functionality, data accessibility, and user experience, MolGlueDB will undergo a comprehensive structural and interface upgrade annually, enhancing data organization, visualization, and overall usability.

By maintaining regular updates and periodic system improvements, MolGlueDB aims to remain a cutting-edge resource for researchers in the field of targeted protein degradation via MGD approaches.

## Conclusion

MGDs have revolutionized drug design by providing novel approaches to enable the targeted degradation of previously difficult-to-target proteins. The integration of artificial intelligence in drug design has further accelerated the development of MGDs. However, this field remains in its developmental stage and requires substantial reliable data to advance. MolGlueDB continues to receive updates and support, now featuring 1629 MGD molecules in the database, which encompasses 1840 relevant entries. This significant increase in data volume enhances the database’s utility. Given the challenges associated with the druggability of MGDs, we have also increased the inclusion of pharmacokinetic data. These enhancements are expected to make MolGlueDB a more valuable resource for the rational design of MGDs. This platform’s capacity to deliver comprehensive data—including physicochemical properties, biological activity profiles, and graphical representations—supports informed decision-making in drug development and research. Ultimately, MolGlueDB serves as a critical resource for advancing scientific understanding and fostering innovations in medicinal chemistry related to MG compounds.

## Supplementary Material

gkaf811_Supplemental_File

## Data Availability

MolGlueDB is freely accessible at https://www.molgluedb.com.
