# Supplementary material for: MolGlueDB: an online database of molecular glues
Source: Nucleic Acids Res. 2025 Aug 21;54(D1):D1510–8. doi: 10.1093/nar/gkaf811 (PMC12807675; doi:10.1093/nar/gkaf811)
Supplement: gkaf811_Supplemental_File [file gkaf811_supplemental_file.docx]

SUPPLEMENTARY DATA

MolGlueDB: An Online Database of Molecular Glues

Xiao Wang^#,1,3^, Zhiyao Zhuang^#,1^, Chengwei Zhang^1^, Bowen Zhang^1^, Wei Zhan^1^, Yifan Wang^1^, Zhaojuan Liu^1^, Shanwen Yuan^1^, Wenjia Niu^1^, Qi He^1^, Yanqing Tian^1^, Ximing Xu*^,1,4^, Senbiao Fang*^,3,4^ and Chong Qin*^,1,2,3,4^

^1^ Key Laboratory of Marine Drugs, Chinese Ministry of Education, School of Medicine and Pharmacy, Ocean University of China, Qingdao, Shandong, 266003, China

^2^ Laboratory for Marine Drugs and Bioproducts, Qingdao Marine Science and Technology Center, Qingdao, Shandong, 266137, China

^3^ Center for Targeted Protein Degradation and Drug Discovery, Ocean University of China, Qingdao, Shandong, 266003, China

^4^ Marine Biomedical Research Institute of Qingdao, Qingdao, Shandong, 266071, China

* To whom correspondence should be addressed. Email: qc@ouc.edu.cn

Correspondence may also be addressed to Ximing Xu. Email: xuximing@ouc.edu.cn

Correspondence may also be addressed to Senbiao Fang. Email: fangsenbiao@gmail.com

^#^ The first two authors should be regarded as Joint First Authors.

**Table S1.** Summary of the data field and type compiled in MolGlueDB.

| **General information of MGDs** | | | | |
| --- | --- | --- | --- | --- |
| Compound ID and Name | 2D structures | IUPAC name | | Therapeutic usage |
| Canonical SMILES | Std. InChl | Std. InChIKey | | Reference |
| Pharmacophore | Core chemical groups | Research stages | | Molecular formula |
|  | | | | |
| **Biological Assay and Experiment data of MGDs** | | | | |
| Experimental logP | Experimental logD | Experimental logS | | Kinetic solubility |
| Thermodynamic solubility | Proteins recruited | Recruiting protein affinity | | Recruiting protein UniProt |
| Active status | | Mode of action | | |
| PrimaryTarget | PrimaryTarget degradative information | | | |
| SecondaryTarget | SecondaryTarget degradative information | | | |
| Ternary EC_50_ | Degron type | Anti-cell proliferation | | Western Blot |
| Pharmacokinetics (PK) | ADMET profile | Safety pharmacology | | *In vivo* pharmacodynamics (PD) |
| Proteomics | Crystal structure (PDB ID) | Cryo-EM ID | |  |
|  | | | | |
| **Calculated physicochemical properties of MGDs** | | | | |
| Molecular weight | Exact mass | | clogP: predicted octanol/water partition | clogS: predicted aqueous solubility |
| tPSA: topological polar surface area | HBDcount: hydrogen bond donor count | | HBAcount: hydrogen bond receptor count | RotatableBondCount: number of rotatable bonds |
| Aliphatic ring count | Aromatic ring count | | Aliphatic hetero ring count | Aromatic hetero ring count |
| Heavy atoms number | Hetero atoms number | | Spiro atoms number | Bridgehead atoms number |

This table summarizes all data categories curated within MolGlueDB, covering (**i**) general molecular information such as compound identifiers, structural representations, and therapeutic usage; (**ii**) biological assay and experimental data encompassing degradation profiles, binding affinities, pharmacological properties, proteomics, and structural data; and (**iii**) calculated physicochemical parameters generated via RDKit.


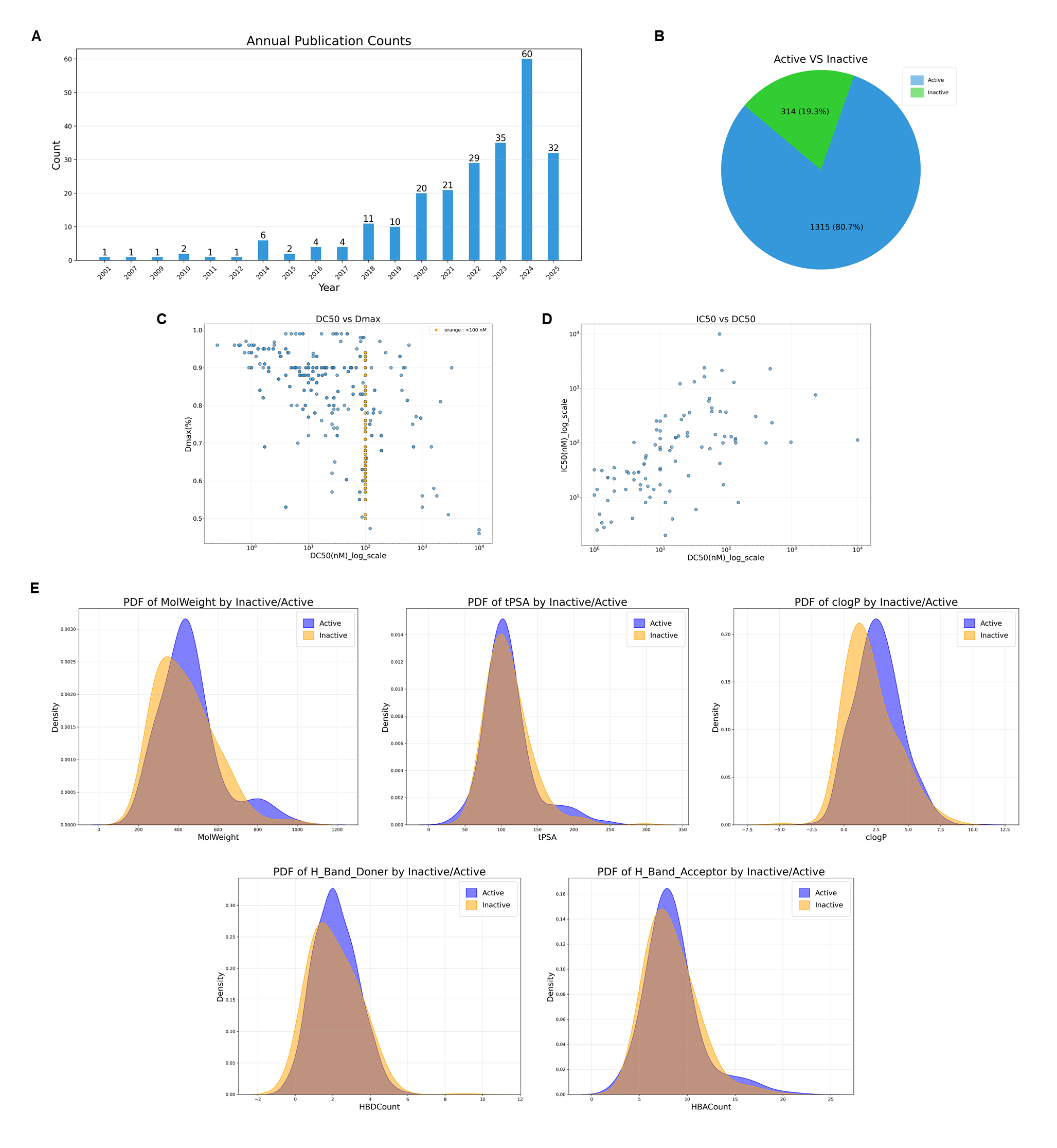


**Figure S1.** Summary statistics of compounds included in MolGlueDB. (**A**) Annual publication counts of MGD-related research articles from January 2001 to May 2025. (**B**) Pie chart showing the proportion of active *vs.* inactive MGDs included. (**C**–**D**) Scatter plots of degradation and antiproliferation data for selected MGDs: (**C**) log_10_DC_50_ *vs.* D_max_, and (D) log_10_DC_50_ *vs.* log₁₀IC_50_. (E) Density function plots of key Lipinski’s Rule of Five parameters for MGDs, including molecular weight (MolWeight), topological polar surface area (tPSA), calculated logP (clogP), and counts of hydrogen bond donors (HBDCount) and acceptors (HBACount).


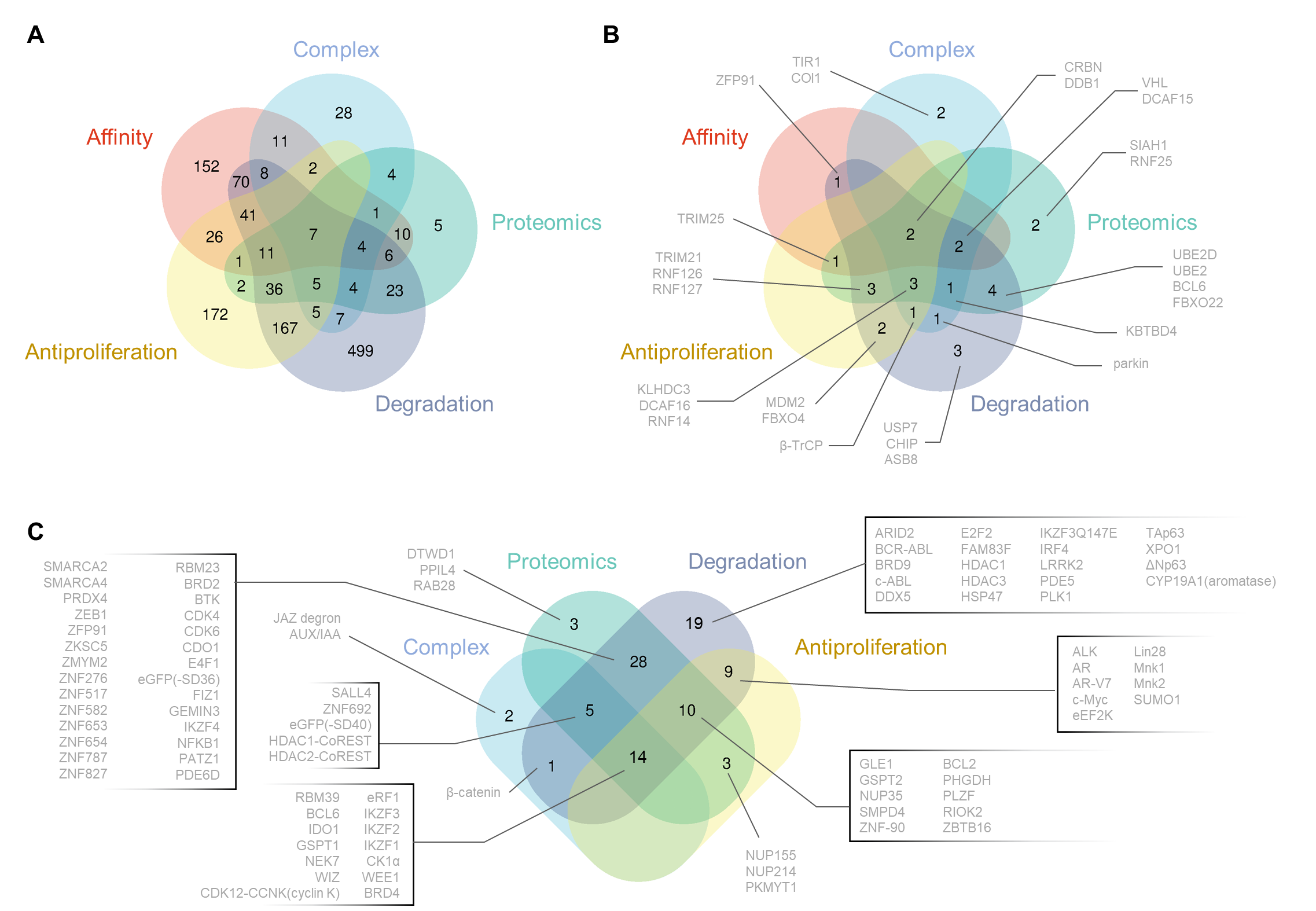


**Figure S2.** Multi-dimensional data coverage analyses in MolGlueDB. The Venn diagrams illustrate the overlap among supporting data categories: Affinity (binding affinity to recruiting protein), Complex (PDB/EMDB structural data), Proteomics (proteomics images), Degradation (Western blot or degradation data), and Antiproliferation (cellular antiproliferation data). Numbers indicate the count of distinct entities within each subset. (**A**) Distinct MGD-based analysis. (**B**) Analysis based on recruiting proteins, with specific proteins labeled and connected to their corresponding regions. (**C**) Analysis based on targets, showing a four-dimensional Venn diagram (excluding recruiting protein affinity) depicting overlaps among Complex, Proteomics, Degradation, and Antiproliferation data. Specific targets within each subset are labeled accordingly.

**
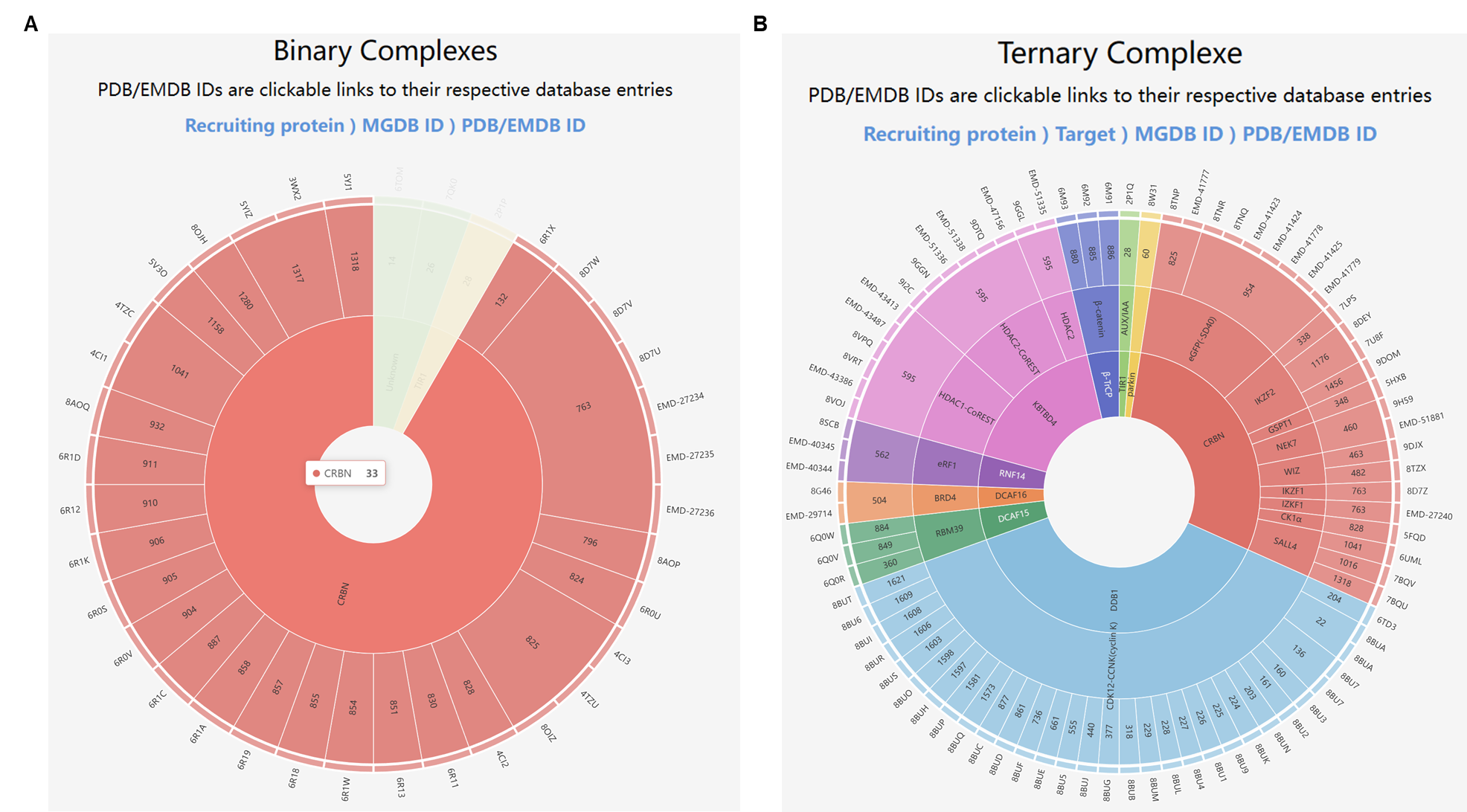
**

**Figure S3.** Interactive sunburst charts implemented using ECharts JavaScript library. These highly interactive visualizations are integrated into the Complexes page of MolGlueDB. Hovering over segments displays the corresponding count of final collected PDB and EMDB IDs. Clicking on rings interactively zooms into specific layers, while clicking directly on PDB or EMDB IDs redirects users to the corresponding RCSB PDB or EMDB server page for detailed viewing. (**A**) Summary of binary complex structures with three hierarchical levels: Recruiting protein → MolGlueDB ID → PDB/EMDB ID. (**B**) Summary of ternary complex structures with four hierarchical levels: Recruiting protein → Target → MolGlueDB ID → PDB/EMDB ID.

***Compound Information Overview Interface***

The MolGlueDB compound information page is structured into six key modules:

1. Compound – Displays essential details such as compound ID, chemical structure, and classification.

2. Pharmacophore – Highlights core structural elements, including commonly used pharmacophores like glutarimide.

3. Primary and Secondary Targets – Lists the primary/secondary target protein(s).

4. Crystal or Cryo-EM Structures – Provides resolved structural data with PDB or EMDB identifiers. When available, the Mol* plugin is embedded to load PDB data directly from the RCSB server, enabling users to interactively examine the detailed structures of the complexes.

5. Recruiting Protein Affinity – Summarizes binding affinity to the recruiting protein.

6. Research Stages – Indicates the clinical development phase, from Discovery to FDA approval.

***Detailed Compound Information Pages***

To enhance usability, clicking on a compound’s "detail" button in the datasheet redirects users to its detailed information page, where all relevant data is consolidated. These pages feature seven specialized tabs:

1. Summary – A high-level overview of the compound.

2. Representations – Displays molecular structures and visual representations.

3. Calculated Properties – Includes calculated physicochemical parameters such as molecular weight, logP, logD, logS, and tPSA.

4. Target Information – Lists all known targets along with degradation data.

5. Experimental Physicochemical Properties – Compiles measured experimental values for solubility, logP, logD, and other key metrics.

6. Activity Data – A repository of biological activity data, covering degradation capabilities, recruiting protein affinity, anti-proliferation, ternary EC_50_, ADMET profile, safety pharmacology, and in vivo PD efficacy values. Graphical representations, such as Western Blot and proteomics figures, are also included to visually demonstrate the compound’s protein degradation efficiency.

7. More Information from Other Articles – Provides external references linking to related studies and databases.
